# Reasons for Ethnic Disparities in the Prehospital Care Pathway Following an Out-of-Hospital Cardiac Event: Protocol of a Systematic Review

**DOI:** 10.2196/40557

**Published:** 2023-07-12

**Authors:** Rochelle Newport, Corina Grey, Bridget Dicker, Shanthi Ameratunga, Matire Harwood

**Affiliations:** 1 Department of General Practice and Primary Health Care Faculty of Medical and Health Sciences University of Auckland Auckland New Zealand; 2 Section of Epidemiology & Biostatistics School of Population Health University of Auckland Auckland New Zealand; 3 Health New Zealand Auckland New Zealand; 4 Paramedicine Department Auckland University of Technology Auckland New Zealand; 5 Clinical Audit and Research St John New Zealand Auckland New Zealand; 6 Counties Manukau Health Auckland New Zealand

**Keywords:** health equity, ethnicity, Indigenous peoples, out-of-hospital cardiac arrest, emergency responders, health care, patient care, cardiovascular, cardiology

## Abstract

**Background:**

Substantial inequities in cardiovascular disease occur between and within countries, driving much of the current burden of global health inequities. Despite well-established treatment protocols and clinical interventions, the extent to which the prehospital care pathway for people who have experienced an out-of-hospital cardiac event (OHCE) varies by ethnicity and race is inconsistently documented. Timely access to care in this context is important for good outcomes. Therefore, identifying any barriers and enablers that influence timely prehospital care can inform equity-focused interventions.

**Objective:**

This systematic review aims to answer the question: Among adults who experience an OHCE, to what extent and why might the care pathways in the community and outcomes differ for minoritized ethnic populations compared to nonminoritized populations? In addition, we will investigate the barriers and enablers that could influence variations in the access to care for minoritized ethnic populations.

**Methods:**

This review will use Kaupapa Māori theory to underpin the process and analysis, thus prioritizing Indigenous knowledge and experiences. A comprehensive search of the CINAHL, Embase, MEDLINE (OVID), PubMed, Scopus, Google Scholar, and Cochrane Library databases will be done using Medical Subject Headings terms themed to the 3 domains of context, health condition, and setting. All identified articles will be managed using an Endnote library. To be included in the research, papers must be published in English; have adult study populations; have an acute, nontraumatic cardiac condition as the primary health condition of interest; and be in the prehospital setting. Studies must also include comparisons by ethnicity or race to be eligible. Those studies considered suitable for inclusion will be critically appraised by multiple authors using the Mixed Methods Appraisal Tool and CONSIDER (Consolidated Criteria for Strengthening the Reporting of Health Research Involving Indigenous Peoples) framework. Risk of bias will be assessed using the Graphic Appraisal Tool for Epidemiology. Disagreements on inclusion or exclusion will be settled by a discussion with all reviewers. Data extraction will be done independently by 2 authors and collated in a Microsoft Excel spreadsheet. The outcomes of interest will include (1) symptom recognition, (2) patient decision-making, (3) health care professional decision-making, (4) the provision of cardiopulmonary resuscitation, (5) access to automated external defibrillator, and (6) witnessed status. Data will be extracted and categorized under key domains. A narrative review of these domains will be conducted using Indigenous data sovereignty approaches as a guide. Findings will be reported according to the PRISMA (Preferred Reporting Items for Systematic Reviews and Meta-Analyses) 2020 guidelines.

**Results:**

Our research is in progress. We anticipate the systematic review will be completed and submitted for publication in October 2023.

**Conclusions:**

The review findings will inform researchers and health care professionals on the experience of minoritized populations when accessing the OHCE care pathway.

**Trial Registration:**

PROSPERO CRD42022279082; https://tinyurl.com/bdf6s4h2

**International Registered Report Identifier (IRRID):**

PRR1-10.2196/40557

## Introduction

Cardiovascular diseases (CVDs) are the leading cause of mortality and morbidity globally [1,2]. However, the burden of disease is distributed inequitably between and within countries [3], contributing to the double burden of disease in low- and middle-income countries [4] and widening ethnic inequities in high-income countries [5-7]. Reducing the burden of CVDs will require a multipronged approach: reducing the exposure to the risk factors for disease as well as optimizing equitable access to existing treatment options [8,9].

Despite advances made in the treatment and prevention of CVDs, ethnic inequities remain. The widespread health inequities experienced by Indigenous populations and other ethnic groups in colonial nations [10,11] are driven by the differential exposure to the social determinants of health [12], racism [13-16], and the ongoing impacts of colonization [17,18]. As such, achieving health equity becomes an ethical imperative, underpinned by a fundamental right to health [19,20] and the understanding that some groups have experienced barriers to realizing these rights.

Addressing inequities in health requires a broad understanding of both the etiology and treatment of disease and the complex interplay between the social determinants of health. Further, the dominant attention on health behaviors and biological responses must be replaced by greater emphasis and critical appraisal of the root causes of inequitable health outcomes, particularly the effects of institutional racism and societal institutions [16] have on health outcomes. Lifting the research gaze further upstream provides the necessary information to inform interventions that will reduce ethnic health inequities. One area within the CVD care pathway that requires further exploration is access to prehospital care following an out-of-hospital cardiac event (OHCE).

The timeliness of care is crucial for positive outcomes. Every 30-minute delay to reperfusion (the restoration of blood flow to a damaged heart) following an ST-elevation myocardial infarction is associated with a 7.5% increased risk of death, and every 10-minute delay is associated with an additional 3.31 per 100 deaths [21]. The timeliness of care is even more important following a cardiac arrest, with the likelihood of survival increasing significantly if bystander cardiopulmonary resuscitation (CPR), defibrillation, or both is performed before emergency medical staff (EMS) arrive [22]. Within this field of research, differential access to care by ethnicity is well-documented [23], particularly following a cardiac arrest. However, no systematic review or attempt to map the care pathway has been undertaken for all nontraumatic, acute OHCEs.

This review will be undertaken as part of the Manawataki Fatu Fatu for Achieving Cardiovascular Care for Equity Studies (ACCESS) program [24], which has a specific focus on achieving equity for Māori and Pacific Islander peoples in Aotearoa, New Zealand. The initial scoping for this review identified that CVD inequities exist between ethnic groups in Aotearoa, New Zealand, with significantly higher CVD hospitalization and mortality rates among Māori (the Indigenous people of New Zealand) and Pacific Islander peoples compared with New Zealand European populations [25-27]. Poorer access to care for Māori and Pacific Islander peoples is likely to be an important contributor to these established inequities [28-30]. There is very little research about the experience of the care pathway for Māori and Pacific Islander peoples following an OHCE. What is known is that despite the higher burden of disease, Māori and Pacific Islander peoples experience delays to treatment [31] and barriers to CPR and defibrillation in the community [28,32]. Importantly, there appears to be limited (1) published literature in New Zealand and (2) opportunities to learn from the experiences of similar communities (ethnic minority groups) internationally. Therefore, a wider review, beyond New Zealand, was preferred.

The objective of this review is to identify the barriers and enablers to care for minoritized populations following an OHCE. The review also seeks to describe the OHCE care pathway that different ethnic groups experience, from the onset of symptoms until the receipt of care from a registered health professional. Within the review, prioritization will be given to research that illustrates the experience of the care pathway for Indigenous communities.

## Methods

### Overview

A pragmatic approach to this systematic review [33] will be undertaken. The overall research question seeks to simplify a complex interaction of diverse populations, systems, and variables that operate in the prehospital cardiac care space. Within this context, the review seeks to determine the service and system-level variables that affect a patients’ journey along the care pathway. For the purposes of this review, the working definition of OHCE will be broad and includes (but is not limited to) cardiac arrest, myocardial infarction, arrhythmia, and chest pain.

### Objectives

This systematic review aims to answer the question:

Among adults who experience an OHCE, to what extent and why might the care pathways in the community differ for minoritized populations compared with nonminoritized populations?

The systematic review also aims to:

- Identify the barriers and enablers that could influence variations in the access to care for minoritized ethnic populations
- Describe what the care pathway in the community looks like for minoritized ethnic populations after an acute cardiac event compared to nonminoritized populations

### Search Strategy

The search strategy, developed in consultation with a specialist librarian, will use a number of medical literature databases and search engines to identify literature for review. The sources include:

- CINAHL
- Embase
- MEDLINE (OVID)
- PubMed
- Scopus
- Google Scholar
- Cochrane Library

Different combinations of Medical Subject Headings (MeSH) terms and keywords related to ethnicity and race, equity, and CVD will be used. During the initial scoping for this review, a very limited number of articles related to the out-of-hospital care pathway in New Zealand were identified. The research question and therefore the search terms for the population of interest were broadened to include other minoritized ethnic communities globally. Although the scope of the review was expanded, the review will be underpinned by Kaupapa Māori research, which seeks to validate Māori experiences in the context of a colonized society [34]. Therefore, the MeSH terms for Māori and Pacific Islander peoples were added as specific search terms in recognition of this. The MeSH terms for Indigenous peoples have been included to capture research specific to other Indigenous or First Nations populations.

As relevant articles are identified, the reference lists will be checked for additional studies and reviews. The search will include articles relating to original research that are published in English with no exclusions due to research design or country setting. The date of publication will be limited to between January 1, 2000, and January 1, 2023. Textbox 1 outlines the search terms that will be used.

**Search terms.**

**Setting**

- Prehospital
- First responder
- Ambulance
- Out of hospital
- Emergency medical
- Transportation of patients
- Emergency medical service
- Rescue
- Paramedic
- Emergency medical technician
- Community

**Health condition**

- Cardiac
- Coronary
- Cardiovascular
- Heart attack
- Arrest
- Myocardial infarction
- ST-elevation myocardial infarction (STEMI)
- Non-STEMI (NSTEMI)
- Chest pain
- Arrhythmia
- Ischemia
- Angina
- Acute coronary syndrome

**Context**

- Ethnicity
- Race
- Culture
- Indigenous peoples
- Disparity
- Inequity
- Inequality
- Equity
- Māori peoples
- Pacific Islander peoples

### Inclusion and Exclusion Criteria

Textbox 2 outlines the inclusion and exclusion criteria that will be used.

**Inclusion and exclusion criteria.**

**Inclusion criteria**

- Outcomes are relevant to the research question
- Publication language: English
- Study population aged >18 years
- Cardiac event is nontraumatic
- Human subjects
- Based in prehospital settings
- All study types

**Exclusion criteria**

- Conference proceedings
- No English translation available
- No full text available
- Study population aged <18 years
- Cardiac event is a result of trauma

### Types of Studies and Settings

All peer-reviewed, original research studies will be considered for inclusion. The “community” setting will include all settings in the prehospital phase, up to arrival at a hospital or emergency medical center. There will be no restriction by country.

### Population

The population is broadly defined as all adults, aged 18 years and older, from minoritized ethnic communities who experience an acute, nontraumatic cardiac event outside of a hospital. The term “minoritized” is purposely chosen to reflect communities whose “collective cultural, economic, political, and social power has been eroded” [35]. The reference group is nonminoritized ethnic populations.

All types of acute cardiac events with a nontraumatic etiology will be considered, including but not limited to cardiac arrest, chest pain, arrhythmia, and myocardial infarction.

### Outcomes

Any type of outcome measures will be included where ethnic and racial specific comparisons within a country are conducted. Analyses may include stratified analyses, the investigation of interactions or effect modification, and comparisons using qualitative data. Outcome measures from multicountry studies will be included if the ethnic groups analyzed are comparable. The primary outcomes will include the analysis of any factors that affect the pathways of care (time, journey, and the process taken to care) and barriers and enablers to prehospital care. This will include outcomes related to comorbidities, health education, symptom recognition, patient and EMS decision-making, care-seeking processes, access to CPR and automated external defibrillator, or informal care received before EMS contact. The secondary outcomes will be any gender analysis by ethnicity and race.

### Critical Appraisal

The first author will undertake the literature search. Identified studies will be collated and managed in an EndNote library created for this review once the studies have been imported to the library. Duplicate entries will be removed before stage 1 of the critical appraisal process. During the initial review process, 2 authors will screen the title and abstract of each article to ensure that papers meet the inclusion criteria.

At stage 2 of the critical appraisal, the full text will be obtained for all articles, and every study will be evaluated by at least 2 authors using the Mixed Methods Appraisal Tool (MMAT) [36]. The MMAT provides a standardized table of quality assessment criteria that can be used for qualitative and quantitative research designs. All studies with Indigenous populations will also be reviewed using the CONSIDER (Consolidated Criteria for Strengthening the Reporting of Health Research Involving Indigenous Peoples) statement [37]. The CONSIDER statement provides a checklist that serves to strengthen Indigenous health outcomes by improving research practice and reporting. Study authors will be contacted to clarify results, resolve any uncertainties, and obtain additional information if needed. Risk of bias will be undertaken using the Graphic Appraisal Tool for Epidemiological studies. Assessment will be done at both study and outcome levels. Articles for inclusion will be identified by all review authors. The included reference lists for included articles will be scanned to identify further articles that may be eligible for inclusion. Articles identified at this stage will also be appraised using the MMAT and CONSIDER statement where appropriate. During the critical appraisal, any disagreement about the inclusion or exclusion of a paper will be resolved by discussion with all review authors.

### Data Extraction

Data for extraction will include general study information, study characteristics, participant characteristics, and study results measured on outcomes identified in this protocol. Appraisals and data extraction will be independently extracted by the authors and recorded in a form. A standardized data extraction form will be used, which will be piloted and refined to ensure that there is consistency between the multiple reviewers. Once finalized, the completed form will populate a Microsoft Excel spreadsheet that will be used for data analysis.

Each eligible study will have data extracted under 4 domains (Textbox 3).

**Extracted domains.**

**General study information**

- Author
- Article title
- Year
- Country

**Study characteristics**

- Aims and objectives
- Study design and type
- Inclusion and exclusion criteria
- Recruitment procedures

**Participant characteristics**

- Sample size
- Ethnicity and race
- Number of male and female participants

**Study results (outcomes)**

- Primary outcomes
  Variables in the care pathway from symptom onset to arrival at a hospital, including but not limited to:
    1. Time, journey, access to cardiopulmonary resuscitation or automated external defibrillator, witnessed by bystander or emergency medical staff (EMS), and informal care before EMS contact
    2. Health outcomes (comorbidities and survival)
    3. Barriers and enablers (health education; decision-making by EMS, patient, or witness; symptom recognition; and care-seeking processes)
- Secondary outcomes
  Any gender analysis by ethnicity and race

### Synthesis of Included Studies

The synthesis of the data will be underpinned by an Indigenous data sovereignty approach that requires researchers to critique how data about Indigenous peoples are collected, used, and analyzed [38,39]. A convergent integrated approach to the data synthesis [40] will be undertaken, whereby quantitative findings will be “qualitized” before conversion to narratives.

It is unlikely that this review will obtain sufficient data that are similar enough to conduct a meta-analysis. It is therefore expected that the authors will conduct a narrative synthesis of the data.

### Reporting

The review findings will be reported according to PRISMA (Preferred Reporting Items for Systematic Reviews and Meta-Analyses) guidelines [41]. The PRISMA 2020 flow diagram will be used to guide the recording of the number of articles identified, screened, included, and excluded.

### Patient and Public Involvement

This is a protocol for a systematic review; therefore, no patients or public will be involved. However, the proposal and approach were informed by discussion with service providers in the prehospital sector in New Zealand. Patient consent for publication is not required.

### Ethical Considerations

Ethics approval is not required for this systematic review as it is reviewing already anonymized, published data.

## Results

Our research is in progress. We anticipate the systematic review will be completed and submitted for publication in October 2023. The findings of this review will be disseminated via a publication in a peer-reviewed journal as well as conference presentations and stakeholder meetings.

## Discussion

### Expected Findings

This paper presents a methodological description of a systematic review process that seeks to inform equity-based intervention and practice. CVD is the leading cause of mortality and morbidity globally. The rates of CVD-related morbidity and mortality have increased, particularly in low- to middle-income countries, [42]. Although population aging contributes to increasing CVD rates globally, barriers to accessing care widens the gap between countries [43] and within countries. For Indigenous and other minoritized ethnic peoples experiencing inequities, identifying the barriers to accessing care is vital for creating culturally grounded interventions that reduce the health inequities they experience [44,45]. This review will contribute to the existing body of knowledge by identifying the factors that improve access to care for different ethnic groups following an OHCE. By integrating an Indigenous quality appraisal tool and research methodology, the review will provide evidence with a uniquely positioned equity lens. It is intended that this will provide insight into areas where interventions can be targeted to increase access to care for ethnic groups in New Zealand and globally. The resulting systematic review will present an unbiased and detailed summary of the current evidence available about the experiences of minoritized populations accessing care following an OHCE compared with nonminoritized populations. The results of this review will inform researchers and emergency service providers on variables related to equity-centered service provision and intervention in the community setting.

### Strengths and Limitations

The strengths of the resulting systematic review include the following:

- This systematic review of factors that facilitate access to care will be based upon a detailed search strategy that includes all study types from any country.
- The review will use a robust reporting tool and a validated quality appraisal checklist for critical appraisal of the literature.
- A social justice and equity lens will be applied to the selection and analysis of identified research.

The limitations of the resulting systematic review include the following:

- By restricting to the English language, this research is limited in its ability to identify articles relevant to other Indigenous people that are published in another language.
- By excluding gray literature, we will not capture the prehospital care reporting mechanisms that are often published as technical reports, for example.
- There is also the risk of publication bias where studies with nonsignificant findings may not be published and therefore will not be captured in our review.

### Conclusion

This systematic review protocol illustrates the transparency of the research and provides a process for conducting a similar review.

## Abbreviations

ACCESS: Achieving Cardiovascular Care for Equity Studies
CONSIDER: Consolidated Criteria for Strengthening the Reporting of Health Research Involving Indigenous Peoples
CPR: cardiopulmonary resuscitation
CVD: cardiovascular disease
EMS: emergency medical staff
MeSH: Medical Subject Headings
MMAT: Mixed Methods Appraisal Tool
OHCE: out-of-hospital cardiac event
PRISMA: Preferred Reporting Items for Systematic Reviews and Meta-Analyses

## Data Availability

The data sets generated and/or analyzed during the current study are available from the corresponding author on reasonable request.

## References

[1] GBD 2017 Causes of Death Collaborators (2018). Global, regional, and national age-sex-specific mortality for 282 causes of death in 195 countries and territories, 1980-2017: a systematic analysis for the Global Burden of Disease Study 2017. Lancet.

[2] GBD 2017 DALYsHALE Collaborators (2018). Global, regional, and national disability-adjusted life-years (DALYs) for 359 diseases and injuries and healthy life expectancy (HALE) for 195 countries and territories, 1990-2017: a systematic analysis for the Global Burden of Disease Study 2017. Lancet.

[3] Yusuf S, Reddy S, Ounpuu S, Anand S (2001). Global burden of cardiovascular diseases: Part II: variations in cardiovascular disease by specific ethnic groups and geographic regions and prevention strategies. Circulation.

[4] Boutayeb A (2006). The double burden of communicable and non-communicable diseases in developing countries. Trans R Soc Trop Med Hyg.

[5] Rabanal KS, Selmer RM, Igland J, Tell GS, Meyer HE (2015). Ethnic inequalities in acute myocardial infarction and stroke rates in Norway 1994-2009: a nationwide cohort study (CVDNOR). BMC Public Health.

[6] Bhopal RS, Humphry RW, Fischbacher CM (2013). Changes in cardiovascular risk factors in relation to increasing ethnic inequalities in cardiovascular mortality: comparison of cross-sectional data in the Health Surveys for England 1999 and 2004. BMJ Open.

[7] Singh GK, Siahpush M, Azuine RE, Williams SD (2015). Widening socioeconomic and racial disparities in cardiovascular disease mortality in the United States, 1969-2013. Int J MCH AIDS.

[8] Joseph P, Leong D, McKee M, Anand SS, Schwalm J, Teo K, Mente A, Yusuf S (2017). Reducing the global burden of cardiovascular disease, part 1: the epidemiology and risk factors. Circ Res.

[9] Leong D, Joseph P, McKee M, Anand S, Teo K, Schwalm J, Yusuf S (2017). Reducing the global burden of cardiovascular disease, part 2: prevention and treatment of cardiovascular disease. Circ Res.

[10] Anderson I, Robson Bridget, Connolly Michele, Al-Yaman Fadwa, Bjertness Espen, King Alexandra, Tynan Michael, Madden Richard, Bang Abhay, Coimbra Carlos E A, Pesantes Maria Amalia, Amigo Hugo, Andronov Sergei, Armien Blas, Obando Daniel Ayala, Axelsson Per, Bhatti Zaid Shakoor, Bhutta Zulfiqar Ahmed, Bjerregaard Peter, Bjertness Marius B, Briceno-Leon Roberto, Broderstad Ann Ragnhild, Bustos Patricia, Chongsuvivatwong Virasakdi, Chu Jiayou, Gouda Jitendra, Harikumar Rachakulla, Htay Thein Thein, Htet Aung Soe, Izugbara Chimaraoke, Kamaka Martina, King Malcolm, Kodavanti Mallikharjuna Rao, Lara Macarena, Laxmaiah Avula, Lema Claudia, Taborda Ana María León, Liabsuetrakul Tippawan, Lobanov Andrey, Melhus Marita, Meshram Indrapal, Miranda J Jaime, Mu Thet Thet, Nagalla Balkrishna, Nimmathota Arlappa, Popov Andrey Ivanovich, Poveda Ana María Peñuela, Ram Faujdar, Reich Hannah, Santos Ricardo V, Sein Aye Aye, Shekhar Chander, Sherpa Lhamo Y, Skold Peter, Tano Sofia, Tanywe Asahngwa, Ugwu Chidi, Ugwu Fabian, Vapattanawong Patama, Wan Xia, Welch James R, Yang Gonghuan, Yang Zhaoqing, Yap Leslie (2016). Indigenous and tribal peoples' health (The Lancet-Lowitja Institute Global Collaboration): a population study. Lancet.

[11] Anderson Ian, Crengle Sue, Kamaka Martina Leialoha, Chen Tai-Ho, Palafox Neal, Jackson-Pulver Lisa (2006). Indigenous health in Australia, New Zealand, and the Pacific. Lancet.

[12] Braveman P, Gottlieb L (2014). The social determinants of health: it's time to consider the causes of the causes. Public Health Rep.

[13] Churchwell K, Elkind MSV, Benjamin RM, Carson AP, Chang EK, Lawrence W, Mills A, Odom TM, Rodriguez CJ, Rodriguez F, Sanchez E, Sharrief AZ, Sims M, Williams O, American Heart Association (2020). Call to action: structural racism as a fundamental driver of health disparities: a presidential advisory from the American Heart Association. Circulation.

[14] Harris R, Tobias M, Jeffreys M, Waldegrave K, Karlsen S, Nazroo J (2006). Effects of self-reported racial discrimination and deprivation on Māori health and inequalities in New Zealand: cross-sectional study. Lancet.

[15] Harris RB, Stanley J, Cormack DM (2018). Racism and health in New Zealand: Prevalence over time and associations between recent experience of racism and health and wellbeing measures using national survey data. PLoS One.

[16] Williams DR, Mohammed SA (2013). Racism and health I: pathways and scientific evidence. Am Behav Sci.

[17] King M, Smith A, Gracey M (2009). Indigenous health part 2: the underlying causes of the health gap. Lancet.

[18] Paradies Y (2016). Colonisation, racism and indigenous health. J Pop Research.

[19] (2007). United Nations Declaration on the Rights of Indigenous Peoples. United Nations.

[20] Braveman P (2006). Health disparities and health equity: concepts and measurement. Annu Rev Public Health.

[21] Scholz K, Maier S, Maier L, Lengenfelder B, Jacobshagen C, Jung J, Fleischmann Claus, Werner Gerald S, Olbrich Hans G, Ott Rainer, Mudra Harald, Seidl Karlheinz, Schulze P Christian, Weiss Christian, Haimerl Josef, Friede Tim, Meyer Thomas (2018). Impact of treatment delay on mortality in ST-segment elevation myocardial infarction (STEMI) patients presenting with and without haemodynamic instability: results from the German prospective, multicentre FITT-STEMI trial. Eur Heart J.

[22] Abrams HC, McNally B, Ong M, Moyer PH, Dyer KS (2013). A composite model of survival from out-of-hospital cardiac arrest using the Cardiac Arrest Registry to Enhance Survival (CARES). Resuscitation.

[23] Shah KSV, Shah ASV, Bhopal R (2014). Systematic review and meta-analysis of out-of-hospital cardiac arrest and race or ethnicity: Black US populations fare worse. Eur J Prev Cardiol.

[24] (2021). Manawataki Fatu Fatu. University of Auckland.

[25] Ministry of Health (New Zealand) (2012). Tupu Ola Moui: Pacific Health Chart Book 2012.

[26] Ministry of Health (New Zealand) (2015). Tatau Kahukura: Māori Health Chart Book 2015, 3rd edition.

[27] Grey C, Jackson R, Wells S, Wu B, Poppe K, Harwood M, Sundborn G, Kerr AJ (2018). Trends in ischaemic heart disease: patterns of hospitalisation and mortality rates differ by ethnicity (ANZACS-QI 21). N Z Med J.

[28] Kerr A, Lee M, Grey C, Pegg T, Fisher N, White H, Nunn C, Williams M, Smyth D, Scott T, Chen R, Zhao J, Tun TR, Harwood M, Devlin G (2019). Acute reperfusion for ST-elevation myocardial infarction in New Zealand (2015-2017): patient and system delay (ANZACS-QI 29). N Z Med J.

[29] Ellison-Loschmann L, Pearce N (2006). Improving access to health care among New Zealand's Maori population. Am J Public Health.

[30] Grey C, Jackson R, Wells S, Randall D, Harwood M, Mehta S, Exeter DJ, Kerr AJ (2016). Ethnic differences in coronary revascularisation following an acute coronary syndrome in New Zealand: a national data-linkage study (ANZACS-QI 12). Heart Lung Circ.

[31] Dicker B, Todd VF, Tunnage B, Swain A, Conaglen K, Smith T, Brett M, Laufale C, Howie G (2019). Ethnic disparities in the incidence and outcome from out-of-hospital cardiac arrest: a New Zealand observational study. Resuscitation.

[32] Dicker B, Garrett N, Wong S, McKenzie H, McCarthy J, Jenkin G, Smith T, Skinner JR, Pegg T, Devlin G, Swain A, Scott T, Todd V (2019). Relationship between socioeconomic factors, distribution of public access defibrillators and incidence of out-of-hospital cardiac arrest. Resuscitation.

[33] Petticrew M, Anderson L, Elder R, Grimshaw J, Hopkins D, Hahn R, Krause Lauren, Kristjansson Elizabeth, Mercer Shawna, Sipe Teresa, Tugwell Peter, Ueffing Erin, Waters Elizabeth, Welch Vivian (2013). Complex interventions and their implications for systematic reviews: a pragmatic approach. J Clin Epidemiol.

[34] Smith LT (1999). Decolonizing Methodologies: Research and Indigenous Peoples.

[35] Selvarajah S, Deivanayagam TA, Lasco G, Scafe S, White A, Zembe-Mkabile W, Devakumar D (2020). Categorisation and minoritisation. BMJ Glob Health.

[36] Hong Q, Pluye P, Fabregues S, Bartlett G, Boardman F, Cargo M (2018). Mixed Methods Appraisal Tool (MMAT), version 2018. Mixed Methods Appraisal Tool.

[37] Huria T, Palmer SC, Pitama S, Beckert L, Lacey C, Ewen S, Smith LT (2019). Consolidated criteria for strengthening reporting of health research involving indigenous peoples: the CONSIDER statement. BMC Med Res Methodol.

[38] Cormack D, Reid P, Kukutai T (2019). Indigenous data and health: critical approaches to 'race'/ethnicity and Indigenous data governance. Public Health.

[39] Walter M, Kukutai T, Taylor J (2016). Data politics and Indigenous representation in Australian statistics. Indigenous Data Sovereignty: Toward an Agenda.

[40] Stern Cindy, Lizarondo Lucylynn, Carrier Judith, Godfrey Christina, Rieger Kendra, Salmond Susan, Apóstolo João, Kirkpatrick Pamela, Loveday Heather (2020). Methodological guidance for the conduct of mixed methods systematic reviews. JBI Evid Synth.

[41] Page MJ, McKenzie JE, Bossuyt PM, Boutron I, Hoffmann TC, Mulrow CD, Shamseer L, Tetzlaff JM, Akl EA, Brennan SE, Chou R, Glanville J, Grimshaw JM, Hróbjartsson Asbjørn, Lalu MM, Li T, Loder EW, Mayo-Wilson E, McDonald S, McGuinness LA, Stewart LA, Thomas J, Tricco AC, Welch VA, Whiting P, Moher D (2021). The PRISMA 2020 statement: an updated guideline for reporting systematic reviews. BMJ.

[42] Roth GA, Mensah GA, Johnson CO, Addolorato G, Ammirati E, Baddour LM, Barengo Noël C, Beaton Andrea Z, Benjamin Emelia J, Benziger Catherine P, Bonny Aimé, Brauer Michael, Brodmann Marianne, Cahill Thomas J, Carapetis Jonathan, Catapano Alberico L, Chugh Sumeet S, Cooper Leslie T, Coresh Josef, Criqui Michael, DeCleene Nicole, Eagle Kim A, Emmons-Bell Sophia, Feigin Valery L, Fernández-Solà Joaquim, Fowkes Gerry, Gakidou Emmanuela, Grundy Scott M, He Feng J, Howard George, Hu Frank, Inker Lesley, Karthikeyan Ganesan, Kassebaum Nicholas, Koroshetz Walter, Lavie Carl, Lloyd-Jones Donald, Lu Hong S, Mirijello Antonio, Temesgen Awoke Misganaw, Mokdad Ali, Moran Andrew E, Muntner Paul, Narula Jagat, Neal Bruce, Ntsekhe Mpiko, Moraes de Oliveira Glaucia, Otto Catherine, Owolabi Mayowa, Pratt Michael, Rajagopalan Sanjay, Reitsma Marissa, Ribeiro Antonio Luiz P, Rigotti Nancy, Rodgers Anthony, Sable Craig, Shakil Saate, Sliwa-Hahnle Karen, Stark Benjamin, Sundström Johan, Timpel Patrick, Tleyjeh Imad M, Valgimigli Marco, Vos Theo, Whelton Paul K, Yacoub Magdi, Zuhlke Liesl, Murray Christopher, Fuster Valentin, GBD-NHLBI-JACC Global Burden of Cardiovascular Diseases Writing Group (2020). Global burden of cardiovascular diseases and risk factors, 1990-2019: update from the GBD 2019 study. J Am Coll Cardiol.

[43] Husain Muhammad Jami, Datta Biplab Kumar, Kostova Deliana, Joseph Kristy T, Asma Samira, Richter Patricia, Jaffe Marc G, Kishore Sandeep P (2020). Access to cardiovascular disease and hypertension medicines in developing countries: an analysis of essential medicine lists, price, availability, and affordability. J Am Heart Assoc.

[44] Nolan-Isles D, Macniven R, Hunter K, Gwynn J, Lincoln M, Moir R, Dimitropoulos Y, Taylor D, Agius T, Finlayson H, Martin R, Ward K, Tobin S, Gwynne K (2021). Enablers and barriers to accessing healthcare services for Aboriginal people in New South Wales, Australia. Int J Environ Res Public Health.

[45] Gall A, Anderson K, Howard K, Diaz A, King A, Willing E, Connolly M, Lindsay D, Garvey G (2021). Wellbeing of Indigenous peoples in Canada, Aotearoa (New Zealand) and the United States: a systematic review. Int J Environ Res Public Health.

